# Metformin modulates the gut microbiome in broiler breeder hens

**DOI:** 10.3389/fphys.2022.1000144

**Published:** 2022-09-14

**Authors:** Emily Van Syoc, Evelyn Weaver, Connie J. Rogers, Justin D. Silverman, Ramesh Ramachandran, Erika Ganda

**Affiliations:** ^1^ Integrative & Biomedical Physiology and Clinical & Translational Sciences Dual-Title PhD Program, The Pennsylvania State University, University Park, PA, United States; ^2^ Department of Animal Science, The Pennsylvania State University, University Park, PA, United States; ^3^ Microbiome Center, The Pennsylvania State University, University Park, PA, United States; ^4^ Center for Reproductive Biology and Health, The Pennsylvania State University, University Park, PA, United States; ^5^ Department of Nutritional Sciences, The Pennsylvania State University, University Park, PA, United States; ^6^ Penn State Cancer Institute, Hershey, PA, United States; ^7^ Department of Statistics, The Pennsylvania State University, University Park, PA, United States; ^8^ Department of Medicine, The Pennsylvania State University, University Park, PA, United States; ^9^ Institute for Computational and Data Science, The Pennsylvania State University, University Park, PA, United States; ^10^ College of Information Science and Technology, The Pennsylvania State University, University Park, PA, United States

**Keywords:** broiler breeder hens, gut microbiome, metformin, polycystic ovary syndrome (PCOS), poultry

## Abstract

Broiler breeder hens, the parent stock of commercial broiler chickens, are genetically selected for rapid growth. Due to a longer production period and the focus of genetic selection on superior carcass traits in their progeny, these hens have the propensity to gain excess adipose tissue and exhibit severe ovarian dysfunction, a phenotype that is similar to human polycystic ovary syndrome (PCOS). Metformin is an antihyperglycemic drug approved for type 2 diabetes that is prescribed off-label for PCOS with benefits on metabolic and reproductive health. An additional effect of metformin treatments in humans is modulation of gut microbiome composition, hypothesized to benefit glucose sensitivity and systemic inflammation. The effects of dietary metformin supplementation in broiler breeder hens have not been investigated, thus we hypothesized that dietary metformin supplementation would alter the gut microbiome of broiler breeder hens. Broiler breeder hens were supplemented with metformin at four different levels (0, 25, 50, and 75 mg/kg body weight) from 25 to 65 weeks of age, and a subset of hens (*n* = 8–10 per treatment group) was randomly selected to undergo longitudinal microbiome profiling with 16S rRNA sequencing. Metformin impacted the microbial community composition in 75 mg/kg metformin compared to controls (adjusted PERMANOVA *p* = 0.0006) and an additional dose-dependent difference was observed between 25 mg/kg and 75 mg/kg (adjusted PERMANOVA *p* = 0.001) and between 50 mg/kg and 75 mg/kg (adjusted PERMANOVA *p* = 0.001) but not between 25 mg/kg and 50 mg/kg (adjusted PERMANOVA *p* = 0.863). There were few differences in the microbiome attributed to hen age, and metformin supplementation did not alter alpha diversity. Bacteria that were identified as differentially relatively abundant between 75 mg/kg metformin treatment and the control, and between metformin doses, included *Ruminococcus* and members of the *Clostridia* family that have been previously identified in human trials of PCOS. These results demonstrate that metformin impacts the microbiome of broiler breeder hens in a dose-dependent manner and several findings were consistent with PCOS in humans and with metformin treatment in type 2 diabetes. Metformin supplementation is a potentially promising option to improve gut health and reproductive efficiency in broiler breeder hens.

## Introduction

Broiler breeder chickens are the progenitors of broiler chickens which are raised for meat production. As such, broiler breeders are genetically selected for carcass traits, fast growth, and low feed conversion ratios. The rapid improvement of agricultural production in the 1900s resulted in a 400% increase in broiler growth from 1957 to 2005 (Zuidhof et al., 2014). Broiler chickens are harvested at 6 weeks of age, but broiler breeder hens have a projected production lifespan of 60 weeks or more. The combination of genetic selection for rapid muscle growth and a longer lifespan than their progeny has resulted in poor reproductive efficiency in broiler breeder hens including decreased egg production and lower fertility and hatchability of eggs (Yu et al., 1992). This phenotype of propensity to accrue excess adipose tissue and severe ovarian dysfunction resembles a condition in humans known as polycystic ovary syndrome (PCOS) (Johnson et al., 2009; Johnson, 2012). Improving reproductive efficiency in broiler breeder hens without feed restriction could improve animal welfare and production value (Decuypere et al., 2010).

Metformin is a synthetic biguanide that is the first-line treatment for type 2 diabetes mellitus (Association, 2011; Bosi, 2009; Federation, W.H.O.I.D, 2006). The known mechanisms of metformin are reduction of hepatic gluconeogenesis, decreased intestinal glucose absorption, and improved insulin sensitivity resulting in increased peripheral glucose uptake (Center for Drug Evaluation and Research, 1995). Furthermore, metformin is the most commonly off-label prescribed drug for the treatment of PCOS (Guan et al., 2020). Multiple systematic reviews have provided evidence that metformin treatment improves reproductive health in women with PCOS, including increased fertilization and pregnancy rates (Maniar et al., 2017), normalization of the endocrine profile, as well as a return to normal menstrual cyclicity (Velazquez et al., 1994; Morin-Papunen et al., 1998; Moghetti et al., 2000; van Santbrink et al., 2005; Xing et al., 2020). In addition to numerous physiologic effects, metformin changes the gut microbiome composition and diversity (Forslund et al., 2015; Wu et al., 2017; Elbere et al., 2020). Metformin is postulated to ameliorate gut microbial dysbiosis that is characteristic of obesity and type 2 diabetes mellitus, potentially shifting the microbiome towards a healthier state (Forslund et al., 2015). Polycystic ovary syndrome is associated with gut microbial dysbiosis, characterized by lower alpha diversity and different beta diversity compared to healthy women, decreased *Akkermensia* and *Ruminococcaceae*, and increased *Bacteroides* and *Escherichia/Shigella*, although the results across studies are varied (Yurtdaş and Akdevelioğlu, 2019). Gut microbial dysbiosis impairs the secretion of β-glucuronidase, an enzyme that deconjugates estrogen and enables binding to estrogen receptors, which in turn decreases circulating estrogen, contributing to reproductive dysfunction including PCOS (Baker et al., 2017). No previous studies have quantified the microbiome in metformin-treated women with PCOS (Rizk and Thackray, 2021), but it is likely that metformin improves symptoms associated with PCOS *via* multiple mechanisms, including improving gut microbial dysbiosis to rescue circulating estrogen levels and subsequent hormonal balance.

Although metformin’s effects on the human gut microbiome have been well-studied, less is known about potential host-microbe-metformin interactions in poultry. Metformin treatment in broiler chicks at 600 mg/kg body weight per day decreased feed intake and body weight, presumably through increased glucagon secretion and appetite suppression (Ashwell and McMurtry, 2003). *In vitro* treatment of broiler breeder hen granulosa cells with metformin decreased the expression of genes related to steroidogenesis and decreased progesterone production, suggesting a potentially beneficial effect of metformin on the reproductive health of broiler breeder hens (Weaver and Ramachandran, 2020). Additionally, the gut microbiome of broiler breeder hens has not been well characterized compared to broiler chicks and laying hens (Kers et al., 2018). The few studies that have assessed the gut microbiome in broiler breeder hens are descriptive, and longitudinal temporal dynamics have not been characterized (Díaz-Sánchez et al., 2019; Trudeau et al., 2020).

To determine if metformin treatment alters the gut microbiome or improves reproductive efficiency in broiler breeder hens, we conducted a trial with four levels of metformin (0, 25, 50, and 75 mg/kg body weight) supplemented in the diet from 25 to 65 weeks of age. A subset of hens (*n* = 8–10/treatment group) was randomly selected for longitudinal profiling of the gut microbiome at 40, 50, and 60 weeks of age *via* high-throughput sequencing of the 16S rRNA gene V4 region. We hypothesized that metformin would modulate the gut microbiome, increase alpha diversity, and decrease the relative abundance of some gram-negative bacteria including *Akkermansia* and *Ruminococcaceae*. We expected these taxonomic changes to accompany improvement in egg laying frequency and production lifespan in the broiler breeder hens that may be driven by host-microbe-metformin interactions.

## Materials and Methods

### Animals and reagents

All animal procedures described herein were approved by Pennsylvania State University’s Institutional Animal Care and Use Committee protocol number PRAMS200746656. A commercial strain of broiler breeder hens (Cobb 500) was maintained at the Poultry Education and Research Center at The Pennsylvania State University (University Park, PA, United States). The chickens were reared according to the Cobb 500 Breeder Management Guide and photo-stimulated beginning at 21 weeks of age. The length of light exposure was increased accordingly as they came into lay and birds were provided with a 16h light:8h dark (4:00 to 20:00) photoperiod for the duration of the study. The broiler breeder hens were moved from the rearing room at 22 weeks of age and randomly allocated to four experimental groups, *n* = 45 hens per treatment group. Broiler breeder hens were housed individually in battery cages and were feed-restricted according to the Cobb Breeder Management Guides and were provided with water *ad libitum*. Supplementation of metformin in the diet (0, 25, 50 or 75 mg/kg body weight; Midwest Veterinary Supply, Lakeville, MN, United States) began at 25 weeks of age and continued through the end of the study at 65 weeks of age. A subset of broiler breeder hens from each treatment group (*n* = 10) were weighed every 10 weeks to adjust the amount of metformin mixed into the feed according to their weight change over time.

### Sample collection

**FIGURE 1:**
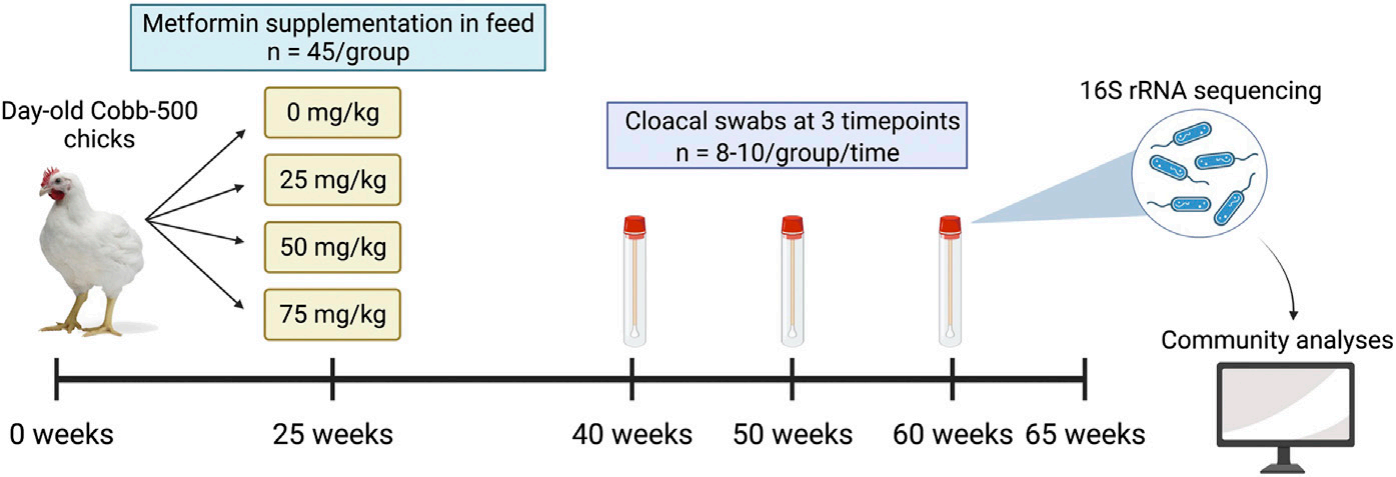
Schematic of experimental design. Broiler breeder hens were raised from day-old chicks and metformin was supplemented in feed at doses of 0, 25, 50, or 75 mg/kg at 25 weeks of age. A subset of hens (*n* = 8–10 per dose) was randomly selected for longitudinal profiling of the microbiome, and cloacal swabs were collected at 40, 50, and 60 weeks of age. The trial ended at 65 weeks of age.

A subset of hens (*n* = 8–10/treatment group) was randomly selected for longitudinal microbiome profiling and cloacal samples were collected at 40, 50, and 60 weeks of age (Figure 1). Sampling timepoints were chosen to coincide with the peak and subsequent decline in egg production. Birds were properly restrained on a breeding stool with chest facing down, and a sterile cotton swab was inserted into the cloaca and angled dorsally and to the right to avoid swabbing the oviduct. Swabs were swirled for 2–3 s, then placed into a sterile 2 ml centrifuge tube and stored on ice until returning to the laboratory, where samples were stored at −80°C until DNA extraction.

### Sequencing library preparation

To optimize extraction yield, 1 ml sterile PBS buffer (pH 7.4) was added to cloacal swabs and homogenized at 20 Hz for 30 min (Bead Ruptor 96, Omni International, Kennesa GA). Swabs were removed and samples were centrifuged at 11,200 rpm for 30 min at 4°C, following which the supernatant was discarded and the pellet re-suspended in 300 µl sterile PBS (pH 7.4). The re-suspended samples were homogenized at 20 Hz for 30 min and stored at −80°C until extraction. High-throughput DNA extraction was performed in a Kingfisher instrument with the MagMAX CORE Nucleic Acid Purification Kit according to manufacturer instructions (Thermo Fisher Scientific, Austin, TX, United States). Extracted DNA quantity and quality were assessed with a spectrophotometer (Nanodrop, Thermo Fisher Scientific Inc., Waltham, MA, United States). Negative and positive controls were extracted alongside the samples and carried through library preparation and sequencing. Genomic DNA was transported on dry ice to Novogene (Sacramento, CA, United States) for high-throughput sequencing of the hypervariable V4 region of the 16S rRNA gene. The sequencing platform was NovaSeq 6000, resulting in 250 × 250 bp paired-end reads.

### Statistical analyses

Adapters were removed and ambiguous bases removed in cutadapt (Martin, 2013). Sequencing quality was visualized with fastQC and MultiQC (Andrews, 2010; Ewels et al., 2016). Quality trimming was performed with Trimmomatic to remove the 20 leading and 20 trailing base pairs, remove reads shorter than 100 bp, and truncate reads at average quality less than 20 in a 4 base pair sliding window (Bolger et al., 2014). Read statistics were collected with seqkit and further pre-processing was conducted in the dada2 R package (Callahan et al., 2016; Shen et al., 2016). Reads were dereplicated in dada2 and the learnErrors function was modified to accommodate binned Illumina quality scores from data generated in Novaseq instruments (see bash script in Data Availability Statement). Paired-end reads were merged and amplicon sequence variants (ASVs) were constructed, after which chimeras were removed with removeBimeraDenovo with the consensus method, and taxonomy was assigned to the genus level with the Silva database v138.1 (Quast et al., 2012). Putative contaminants (ASVs that appeared in negative controls or non-mock-community strains in the positive controls) were removed, non-bacterial ASVs or ASVs unassigned at the phylum level were removed, and ASVs with total relative abundance less than 1e-5 were removed. Negative and positive controls are further discussed in Supplementary Material.

All comparisons were made at the genus level. Statistical analyses comprised three hypothesis tests and subsequent correction for multiple comparisons to assess the longitudinal effects of metformin treatment. To determine an overall effect of metformin supplementation, the 75 mg/kg metformin treatment was compared to 0 mg/kg metformin. To profile the longitudinal effect of metformin, hen age was compared in the metformin-treated groups (combined). To detect a potential dose response, the three metformin doses (25, 50, and 75 mg/kg) were compared (hen age combined). These statistical comparisons were assessed in microbial alpha diversity, beta diversity, and differential relative abundance. Evenness (within-sample or alpha diversity) was calculated as Simpson’s index in the phyloseq R package on filtered count data (McMurdie and Holmes, 2013). Outliers in Simpson’s index were considered as greater/lesser than three times the standard deviation and one outlier was removed. Simpson’s index was tested for normality using a diagnostic residual QQ-plot and residual histogram, and one-way ANOVA or *t*-test was performed. Significance was determined by comparing the raw *p* value to the critical alpha value calculated with Bonferonni’s correction for three comparisons (*α*_critical_ = 0.05/3 = 0.01667). Post-hoc tests were conducted with Tukey’s honest significant differences. Count data was transformed to center log-ratio (CLR) and visualized in a Principal coordinates analysis (PCA) with the microViz package (Gloor et al., 2017; Barnett et al., 2021). Beta diversity was assessed by permutational ANOVA (adonis test) with 999 permutations on Aitchison distances in the microViz package. Significance was determined as described above (*α*_critical_ = 0.05/3 = 0.01667). Differential relative abundance was assessed with linear models on log2-transformed total sum scaled data in the microViz R package, and significance was determined as described above, correcting for 789 hypothesis tests (262–264 bacterial genera in each of three comparisons; *α*_critical_ = 0.05/789 = 6.33e^−5^). Visualizations were made with the microViz, ggplot2, or ggpubr R packages, or BioRender.com (Wickham, 2009; Kassambara, 2020; Barnett et al., 2021).

## Results

### Taxa summary

**FIGURE 2:**
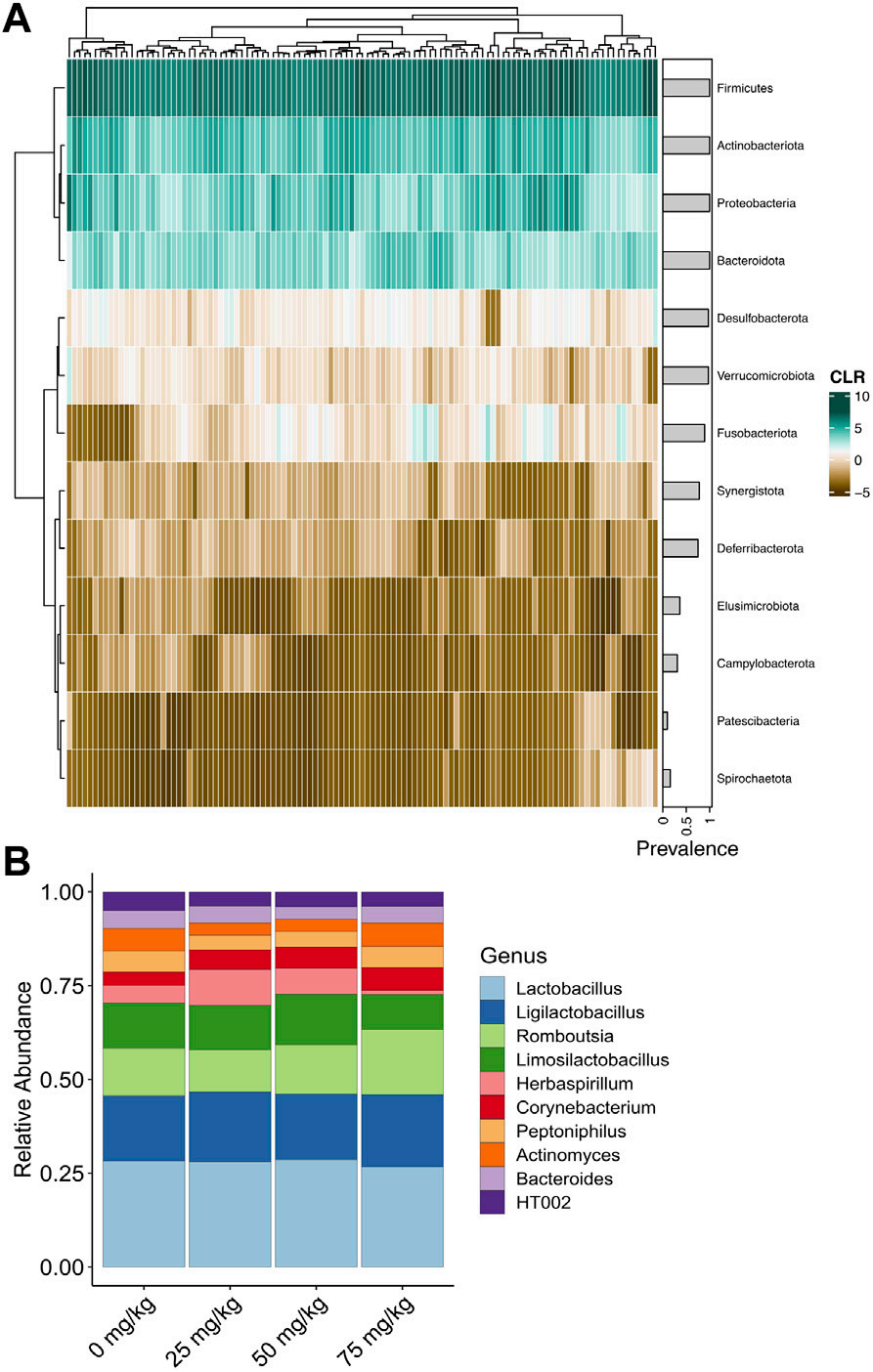
Relative abundance of bacteria detected in the cloaca of broiler breeder hens. **(A)** Heatmap of centered log-ratio relative abundance of bacterial phyla detected in the cloacal swabs of broiler breeder hens. Grey horizontal bars show the prevalence of each phylum. **(B)** Average relative abundance of the top 10 most abundant bacterial genera in each metformin dose.

A total of 18,569,621 raw reads were filtered to 13,486,669 reads with an average of 91,126 reads per sample (Supplementary Table S1). The dada algorithm assigned 24,146 ASVs. After filtering and removing putative contaminants, 274 bacterial genera comprised the final dataset (Supplementary Material). The most abundant and prevalent phylum was Firmicutes, followed by Actinobacteriota, Proteobacteria, and Bacteriodota (Figure 2A; Supplementary Figure S3). The most abundant genera included *Lactobacillus*, *Ligilactobacillus*, *Romboutsia*, *Herbaspirillum*, and *Corynebacterium*, representing the Firmicutes, Proteobacteria, and Actinobacteriota phyla (Figure 2B).

### Alpha diversity

Simpson’s index was not affected by metformin treatment or hen age (Supplementary Table S2). There were no differences between 0 and 75 mg/kg metformin (*t*-test *t* = 1.0683, Bonferonni-adjusted *p =* 0.878), between 40, 50, or 60 weeks of age (one-way ANOVA F_2,82_ = 2.168, Bonferonni-adjusted *p =* 0.368), or between metformin doses (one-way ANOVA F_2,82_ = 1.19, Bonferonni-adjusted *p =* 0.927).

### Beta diversity

**FIGURE 3:**
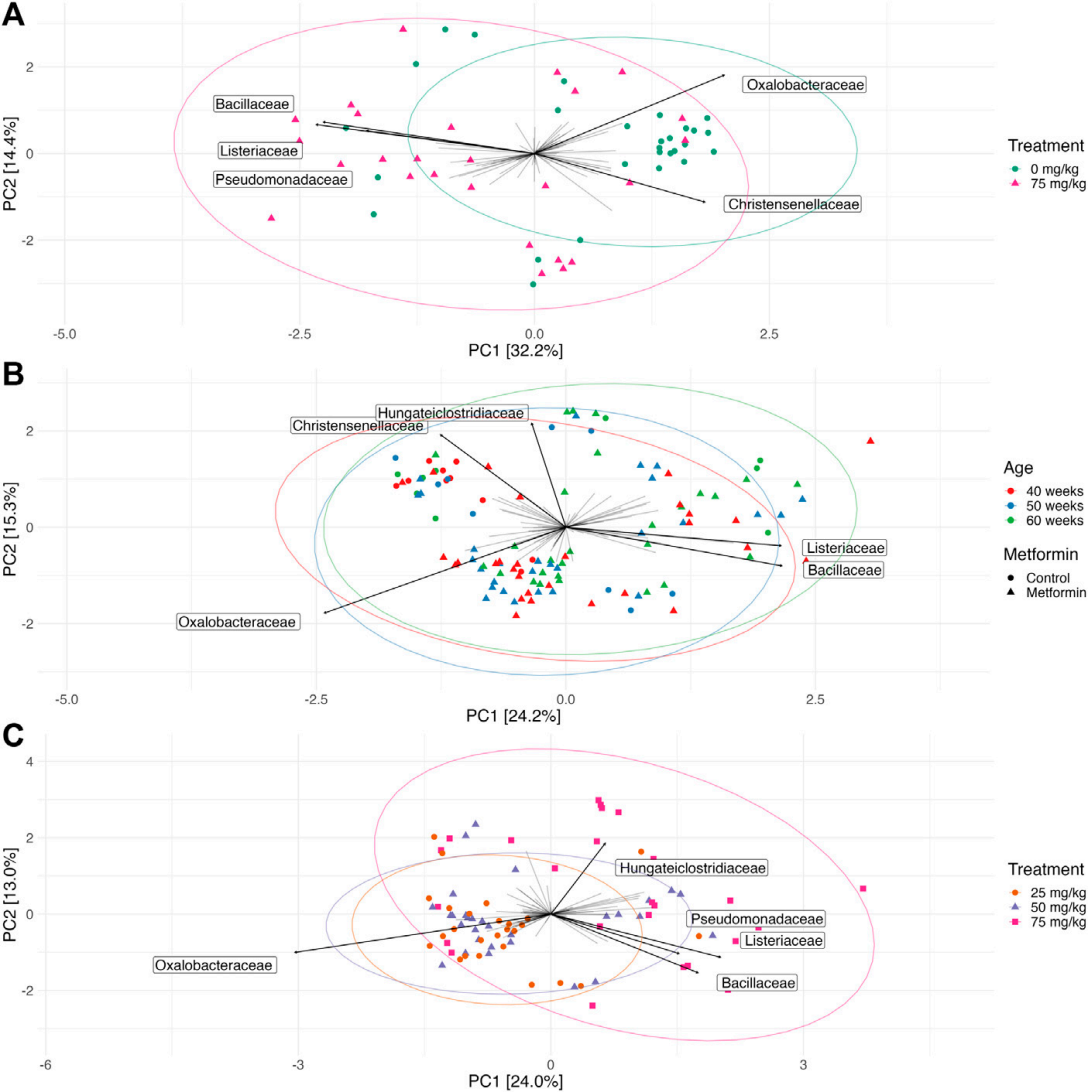
Microbial community composition is affected by metformin dose but not hen age. Principal coordinates analysis (PCA) of center log-transformed microbiome data at the family level showing the top five bacterial family loadings for each ordination. Ellipses represent 95% confidence intervals around the group centroid. **(A)** An overall metformin effect is observed by comparing the microbial community composition 0 mg/kg (purple circles) to 75 mg/kg (green triangles) metformin-treated hens. **(B)** Microbial community composition of broiler breeder hens by age. Colored points show hen age and shapes show metformin treatment (circle for control 0 mg/kg metformin; triangle for 25, 50, and 75 mg/kg metformin). **(C)** Metformin doses impact microbial community structure. (25 mg/kg metformin, green circles; 50 mg/kg metformin, blue triangles; 75 mg/kg metformin, purple squares).

Permutational ANOVA revealed a difference in microbial community structure between the control and 75 mg/kg metformin treatment (Bonferroni-adjusted *p* = 0.0015, *r* ^2^ = 0.06) (Figure 3A). Taxa PCA loadings at the family level indicated that *Oxalobacteraceae* and *Christensenellaceae* were associated with 0 mg/kg metformin while *Pseudomonadaceae*, *Listeriaceae*, and *Bacillaeceae* were associated with 75 mg/kg metformin treatment (Figure 3A). Hen age was not significant after correction for multiple comparisons (Bonferroni-adjusted *p* = 0.3192, *r* ^2^ = 0.032) (Figure 3B). Metformin treatment had a dose-dependent effect on microbial community structure (Bonferroni-corrected *p* = 0.0003, *r* ^2^ = 0.069) and pairwise comparisons showed differences between 25 mg/kg and 75 mg/kg metformin (pairwise adonis *p* = 0.001) and between 50 mg/kg and 75 mg/kg metformin (pairwise adonis *p* = 0.001), but not between 25 mg/kg and 50 mg/kg metformin (pairwise adonis *p* = 0.832) (Figure 3C). Taxa PCA loadings suggested that *Hungateiclostridiaceae*, *Pseudomonadaceae*, *Listeriaceae*, and *Bacillaceae* were associated with 75 mg/kg metformin while *Oxalobacteraceae* was associated with a small cluster of samples belonging to 25 mg/kg and 50 mg/kg metformin (Figure 3C). Metformin doses also differed in group dispersion (beta dispersion *p* = 0.00001) and post-hoc comparisons demonstrated a similar trend to PERMANOVA; there were differences in dispersion between 25 mg/kg and 75 mg/kg metformin (Tukey’s post-hoc *p* = 0.0001) and between 50 mg/kg and 75 mg/kg metformin (Tukey’s post-hoc *p* = 0.0006), but not between 25 mg/kg and 50 mg/kg metformin (Tukey’s post-hoc *p* = 0.99) (Supplementary Figure S4). Hen age was not a significantly confounding factor for either metformin treatment or dose (Supplementary Figure S5A).

### Differential relative abundance

**FIGURE 4:**
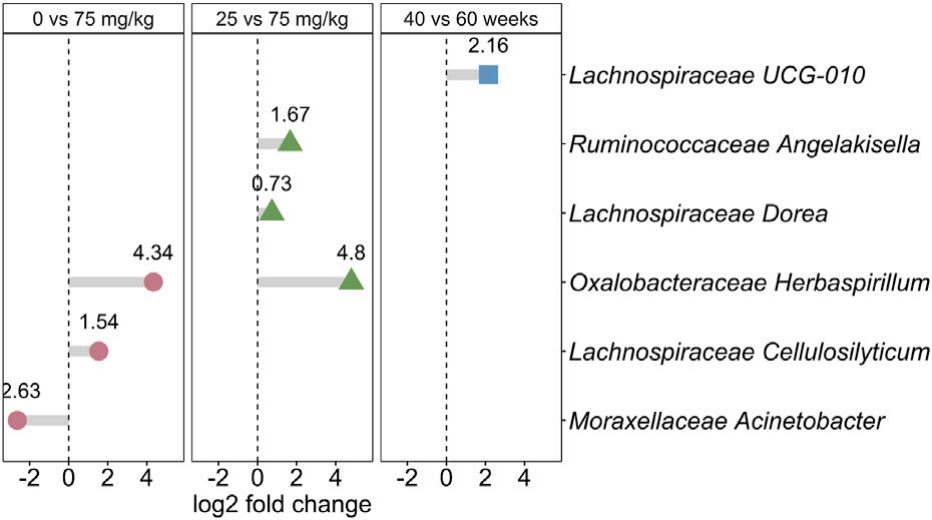
Metformin changes the relative abundance of bacterial genera. The effect size (log2 fold change) is shown for each bacterial genus, shown with the family name, that was significantly different between 0 mg/kg and 75 mg/kg metformin (pink circles; positive log2 fold change is more abundant in 75 mg/kg), between 25 and 75 mg/kg metformin (green triangles; positive log2 fold change in more abundant in 75 mg/kg), or between 40 and 60 weeks of age (blue squares; positive log fold change is more abundant at 60 weeks). The log2 fold change is shown above each point.

The effects of metformin dose and hen age changed the relative abundance of bacterial genera (Figure 4; Supplementary Table S3). Two genera, *Oxalobacteraceae Herbaspirillum* and *Lachnospiraceae Cellulosilyticum*, were significantly more relatively abundant in 75 mg/kg than 0 mg/kg (log2 fold change 4.38 and 1.54, Bonferroni-adjusted *p* = 0.026 and 0.0072, respectively). *Moraxellaceae Acinetobacter* was significantly less abundant in 75 mg/kg compared to 0 mg/kg (log2 fold change −2.63, Bonferroni-adjusted *p* = 0.0038). A dose effect was observed in three genera, shown with the bacterial family, that were all significantly more abundant in 75 mg/kg compared to 25 mg/kg metformin; *Ruminococcaceae Angelakisella* (log2 fold change = 1.67, Bonferroni-adjusted *p* = 0.012), *Lachnospiraceae Dorea* (log2 fold change = 0.73, Bonferroni-adjusted *p* = 0.0337), and *Oxalobacteraeceae Herbaspirillum* (log2 fold change = 4.8, Bonferroni-adjusted *p* = 0.00047). *Herbaspirillum* comprised a larger proportional abundance than the other significant taxa, which were detected in very low abundance in the dataset (Supplementary Figure S6). Only one genus changed throughout the production lifespan of metformin-treated broiler breeder hens. *Lachnospiraceae UCG-010* was more abundant at 60 weeks compared to 40 weeks (log2 fold change 2.16, Bonferonni-adjusted *p* = 0.017). Hen age did not significantly confound the effects of metformin treatment or dosage (Supplementary Figure S5B).

## Discussion

Metformin is an anti-hyperglycemic drug prescribed off-label for the treatment of human polycystic ovary syndrome (PCOS). Broiler breeder hens are genetically selected for fast growth and have reproductive dysfunction that can be phenotypically similar to PCOS. In this study, we supplemented metformin in the feed of broiler breeder hens and profiled the microbiome from 40 to 60 weeks of age to determine if metformin altered the gut microbiome. Metformin was supplemented in the diet at three doses (25, 50, and 75 mg/kg) in addition to a control treatment (0 mg/kg) and the cloacal microbiome was profiled with 16S rRNA sequencing at 40, 50, and 60 weeks of age. We hypothesized that metformin treatment would modulate the gut microbiome, thereby increasing alpha diversity, and decreasing the relative abundance of some gram-negative species. We found that metformin affected the gut microbiome in a dose-dependent manner and there were few significant interactions with hen age.

In contrast with our hypothesis, the effect of metformin on gut microbiome alpha diversity (as measured by Simpson’s index) was not significant. This is consistent with human studies on metformin, which have shown a slight reduction in alpha diversity in healthy people but no effect in type 2 diabetics (Elbere et al., 2020). Although we did not detect an effect of metformin on alpha diversity, there was an effect of metformin on beta diversity (composition of the gut microbiome). We detected significant differences in beta diversity between 0 and 75 mg/kg metformin, and a dose-dependent effect was observed between 25 mg/kg and 75 mg/kg and between 50 mg/kg and 75 mg/kg metformin. This is consistent with much of literature in human metformin treatment in type 2 diabetes, in which metformin exerts a strong effect on the gut microbiome composition as early as 24 h after initial treatment (Forslund et al., 2015; Wu et al., 2017; Sun et al., 2018; Elbere et al., 2020). There were no differences between 25 mg/kg and 50 mg/kg metformin, suggesting that the higher doses had a more noticeable effect on microbial composition. A few bacterial families that may have driven differences between 0 and 75 mg/kg metformin, indicated with PCA taxa loadings, included *Oxalobacteraceae*, *Pseudomonadacea* (Proteobacteria phylum), *Christensenellacea*, *Listeriaceae, and Bacillaceae* (Firmicutes phylum). We did not detect an effect of hen age on beta diversity, suggesting that hen age may not be a large driver of microbial community composition. Furthermore, hen age was not a significant variable in models comparing metformin treatment or dosage for beta diversity and differential relative abundance. Notably, broiler breeder hens have a much longer lifespan than their progeny, which are harvested at approximately 6 weeks, and longitudinal studies to characterize the microbiome are lacking; however, our results are consistent with a previous study that quantified the broiler breeder hen gut microbiome until 16 weeks of age and found that the microbiome stabilized after 3 weeks of age (Díaz-Sánchez et al., 2019).

Previous descriptive studies of the gut microbiome in broiler breeder hens were sampled from aggregated fecal samples collected from the pen but were in general agreement that the microbiome is dominated by Firmicutes, Actinobacteria, and Bacteroidetes (Díaz-Sánchez et al., 2019; Trudeau et al., 2020). We detected 1,270 ASVs and 271 genera in the cloacal swabs, which is similar to a meta-analysis that concluded 915 operational taxonomic units (OTUs) comprising 117 genera were present in the gut microbiome of broiler chickens (Clavijo and Flórez, 2018). Differential relative abundance tests revealed an effect of metformin on the relative abundance of only a few bacterial genera. Two bacterial genera were more relatively abundant in the 75 compared to 0 mg/kg metformin treatment, *Herbaspirillum* and *Cellulosilyticum*. *Cellulosilyticum* is a member of the *Clostridia* class of the Firmicutes phylum, which can be decreased in women with PCOS, but *Herbaspirillum* has not been previously associated with either metformin treatment or metabolic disease (Yurtdaş and Akdevelioğlu, 2019). *Acinetobacter,* a gram-negative coccobacillus of the *Moraxellaceae* family, was more abundant in 0 mg/kg compared to 75 mg/kg metformin. *Acinetobacter* is a potential pathogen and source of antibiotic resistance in humans and has been previously identified in fecal samples of broiler breeder hens, although it is unclear if it contributes to antibiotic resistance in poultry production (Munoz-Price and Weinstein, 2008; Karlsson et al., 2013; Trudeau et al., 2020).

A dose-dependent effect of metformin was observed in three genera that were all most abundant in 75 mg/kg compared to 25 mg/kg metformin; *Angelakisella*, *Dorea*, and *Herbaspirillum. Herbaspirillum* is a member of the *Oxalobacteraceae* family, which was a discriminating taxon loading in the PCA to distinguish both metformin dose and metformin compared to 0 mg/kg control. This suggests that *Oxalobacteraceae* may be strongly affected by metformin treatment with a dose-dependent effect. As neither the *Oxalobacteraceae* nor the genus *Herbaspirillum* have been previously associated with metformin treatment, this may be a species- or environment-specific finding. *Dorea*, a member of the *Lachnospiraceae* family, has been previously observed to be decreased in rodent models of PCOS in addition to associated with metformin treatment (Sun et al., 2018; Zhang et al., 2020; Rizk and Thackray, 2021). *Angelakisella* has not been previously associated with metformin or broiler breeder hens, but the family *Ruminococcaceae* is a well-established marker of dysbiosis that is consistently found to be depleted in irritable bowel disease, colorectal cancer, and human models of PCOS (Wirbel et al., 2019; Yurtdaş and Akdevelioğlu, 2019; Brüssow, 2020; Rizk and Thackray, 2021). Thus, some taxonomic findings seem to be species-specific while others are well-known players of metabolic disease and metformin treatment. While we hypothesized that metformin would affect the relative abundance of *Akkermansia*, such as has been documented in human studies, *Akkermansia* was not among the bacterial genera that was significantly changed by metformin treatment (Rodriguez et al., 2018). This may be because *Akkermansia* is not well documented in chicken microbiomes and may be more specific to the human gastrointestinal tract (Rychlik, 2020).

In addition to an impact on the gut microbiome, metformin supplementation at 75 mg/kg body weight was associated with a significant decrease in the body weight and accretion of abdominal adipose tissue, a normalization of the ovarian follicular hierarchy, a significant increase in the number of eggs laid/hen over the treatment period and an improved plasma endocrine profile of reproductive hormones (Weaver and Ramachandran, 2022). A limitation of this study is that individual correlations between bacterial genera and production metrics were not possible since most production metrics were collected in aggregate. Overall, metformin treatment in broiler breeder hens impacted the gut microbiome composition (beta diversity) but not evenness (alpha diversity) in a dose-dependent manner, and several taxonomic findings were consistent with prior human studies. This suggests that the effects of obesity, PCOS, and metformin are not completely specific to the host species or environment, and that there may be direct effects of metformin on bacterial in the gastrointestinal tract.

We found that the microbial community composition in hens treated with higher doses of metformin (75 mg/kg body weight) were distinguished from lower doses (25 mg/kg and 50 mg/kg body weight). We observed that the gut microbiome did not change throughout the peak and decline of the production cycle, since there were few differences in alpha and beta diversity between 40, 50, and 60 weeks of age. Several bacterial genera were identified that were affected by metformin, including members of the *Clostridia* and *Ruminococcaceae* family which have been implicated in PCOS, type 2 diabetes, and metformin treatment (Karlsson et al., 2013; Wilkins et al., 2019; Rizk and Thackray, 2021). As metformin treatment also resulted in decreased body weight and increased egg production, we hypothesize that metformin-mediated modulation of the gut microbiome may contribute to beneficial shifts in metabolism and reproduction. Furthermore, given the dose effect we observed of only the 75 mg/kg metformin treatment, we postulate that a higher dose of metformin may be necessary to observe microbiome-mediated physiological effects. However, the novelty and relatively small size of this trial precludes drawing strong conclusion and husbandry recommendations. While future research is necessary to unravel the mechanisms underlaying host-microbe-metformin interactions, this study furthers our knowledge of the effects of metformin on the gut microbiome.

## Data Availability

The datasets presented in this study can be found in online repositories. The names of the repository/repositories and accession number(s) can be found below: https://www.ncbi.nlm.nih.gov/bioproject/PRJNA826088, https://github.com/gandalab/broiler-breeder-metformin.
