# Supplementary material for: Metformin modulates the gut microbiome in broiler breeder hens
Source: Front Physiol. 2022 Sep 14;13:1000144. doi: 10.3389/fphys.2022.1000144 (PMC9531308; doi:10.3389/fphys.2022.1000144)
Supplement: Supplementary file 1 [file Table1.docx]

**Supplementary Materials**

**Metformin modulates the gut microbiome in broiler breeder hens**

Van Syoc et al., 2022

**Supplementary Methods 1: Microbiome negative and positive controls.**

Three negative controls containing lysis buffer from the MagMAX Core Microbiome Nucleic Acid Extraction Kit were added to the DNA extraction protocol alongside the samples and carried through library preparation and sequencing. The negative controls contained a median of 117,709 raw reads which decreased to 94,142 reads after quality filtering. Of the 24,146 ASVs in the dataset, 1,577 were detected in the negative controls and removed from the dataset.

Three mock communities of microbial cells that were added to the DNA extraction protocol alongside samples and carried through library preparation and sequencing (ZymoBiomics Community Standard #D6300). The positive controls contained a median of 118,030 raw reads which decreased to 85,202 reads after processing. Of the 24,146 ASVs in the dataset, 1,375 were detected in the positive controls. The mock community contained eight strains of known relative abundance, so all ASVs not assigned to the eight “true” taxa were removed.

**Supplementary Figure 1: Taxonomic composition (at Phylum level) of negative controls.**

**
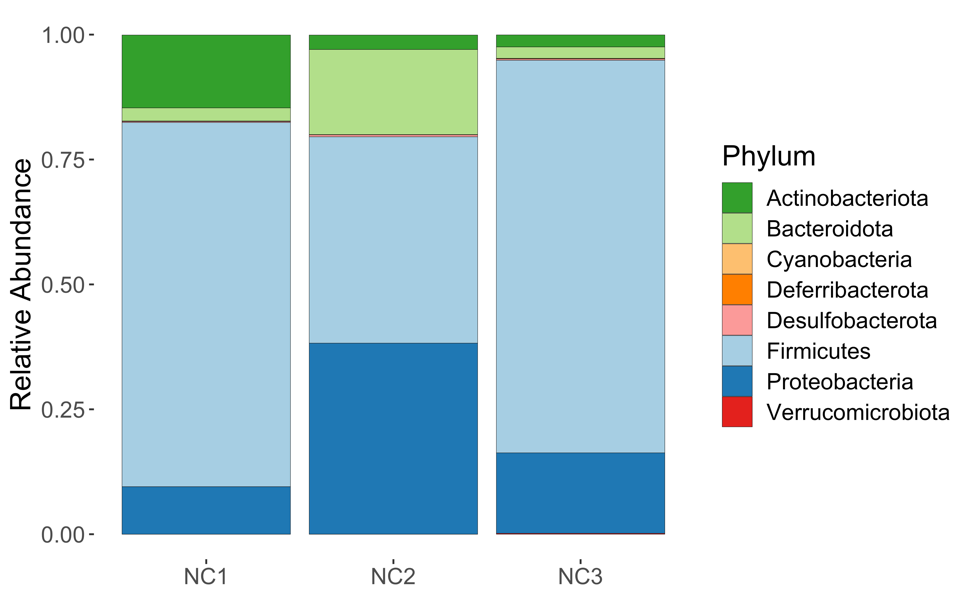
**

**Supplementary Figure 2: Taxonomic composition of positive controls (mock communities) compared to the expected values (“Known”) after removing putative contaminants. The relative abundance of strains in the mock community are expected to vary up to 15%.**

**
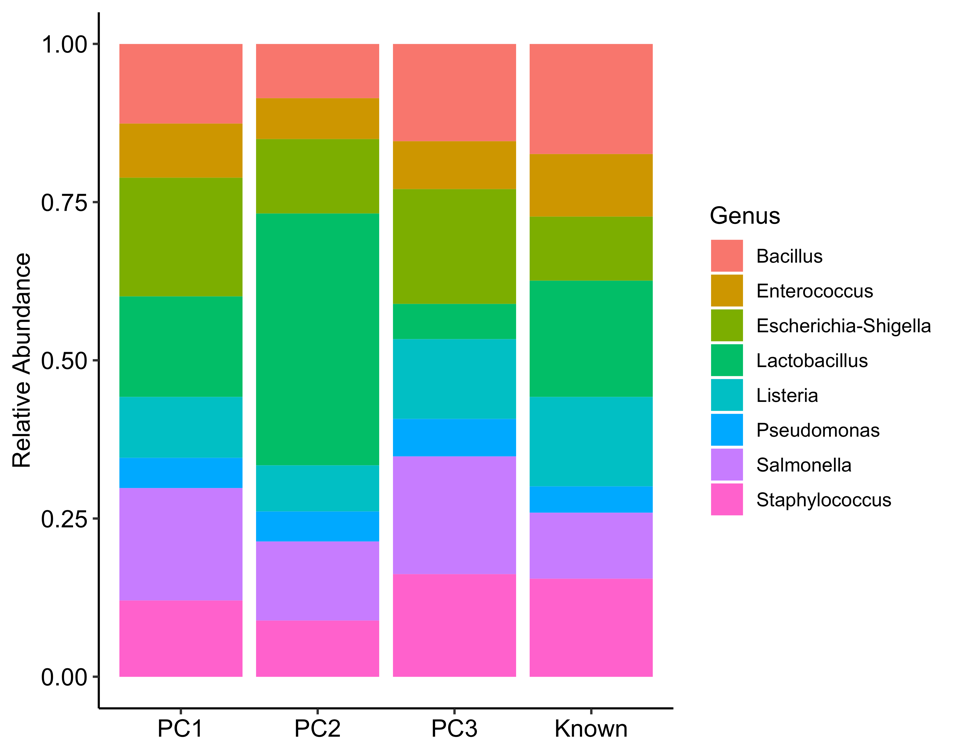
**

**Supplementary Figure S3: Relative abundance of the cloacal microbiome is dominated by the *Firmicutes* phylum.**

**
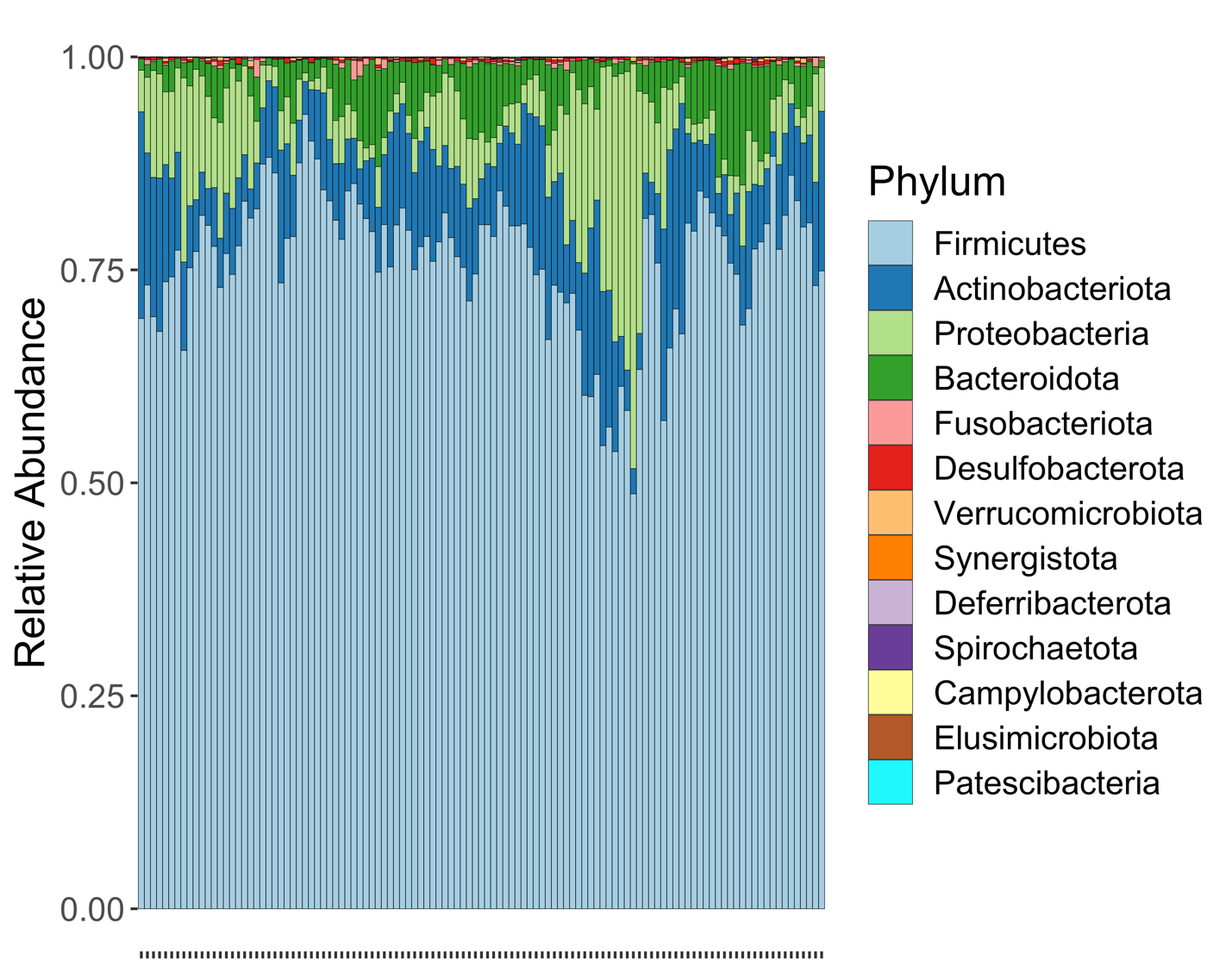
**

**Supplementary Figure S4: Multivariate variance homogeneity visualized as beta dispersion on Aitchison’s distance. The labels indicate the group centroid and points show individual samples and their distance from the group centroid.**

**
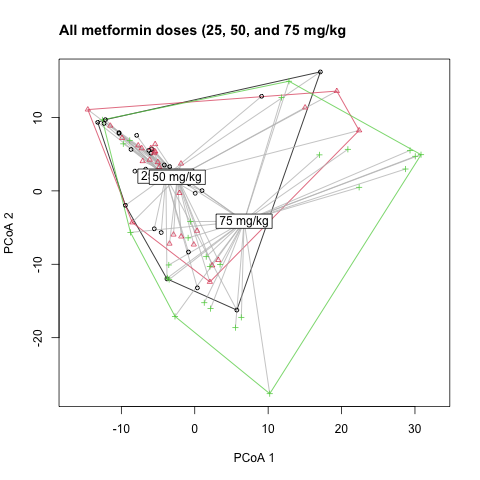
**

**Supplementary Figure S5: To ensure that effects attributed to metformin treatment and dose were not confounded by hen age, “age” was added as a variable to the PERMANOVA (comparison for beta diversity) and log2-transformed linear model (comparison for differential relative abundance). Alpha diversity was not significant for any comparisons so it was excluded from this analysis. (A) The table shows the effect size, r^2^, and the Bonferroni-adjusted *P* value of metformin treatment and dose comparisons for beta diversity with the model including hen age. (B) The figure reflects the results of the linear model with hen age added. The direction and magnitude of all effect sizes were unchanged. One genera, *Lactobacillaceae Limosilactobacillus*, was found to be significantly affected by age in the 0 vs 75 mg/kg test, albeit with a small effect size.**

**A.**

| **Comparison** | **PERMANOVA r^2^** | **Adjusted *P*** |
| --- | --- | --- |
| 75 vs 0 mg/kg metformin | 0.063 | 0.0015 |
| Metformin dose (75, 50, 25 mg/kg) | 0.070 | 0.0001 |

**B.**

**
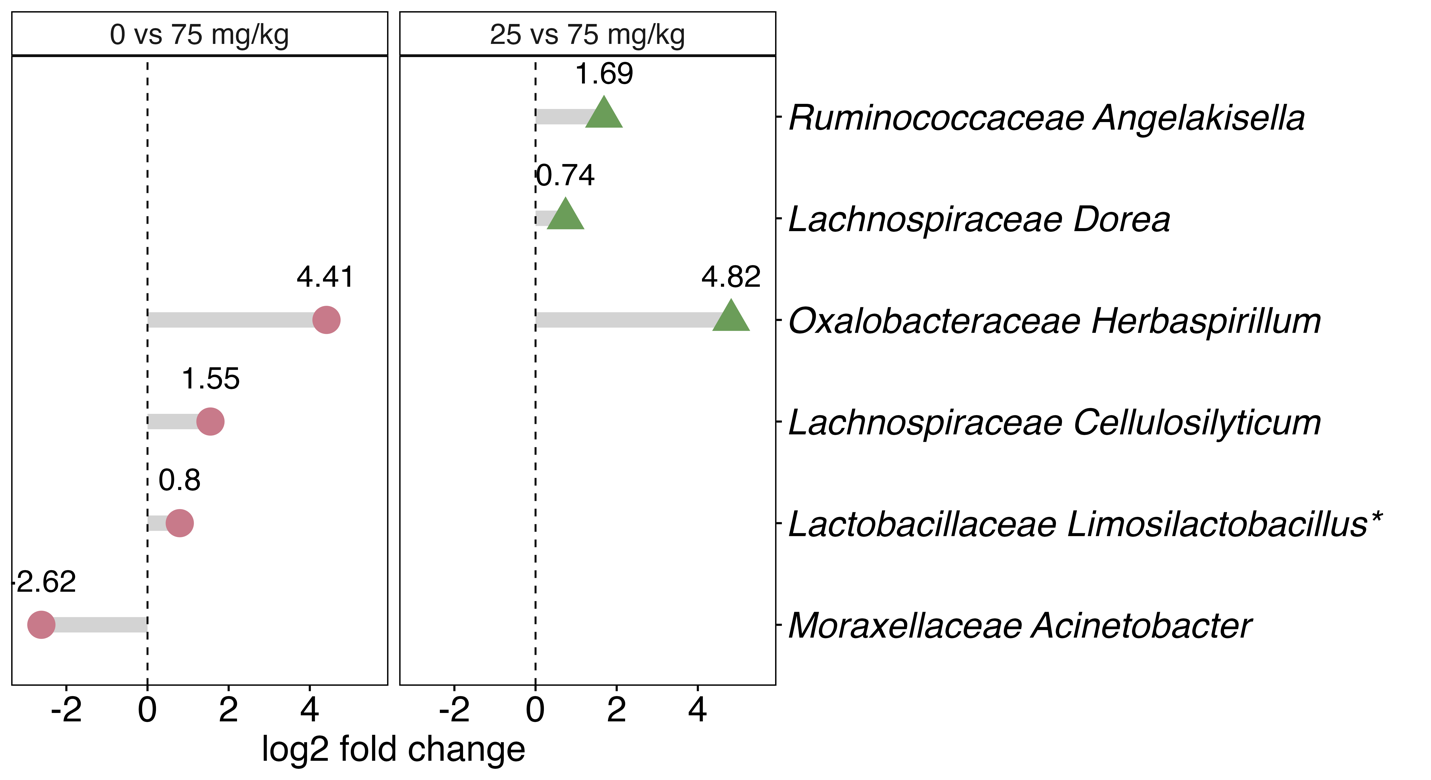
**

**Supplementary Figure 6: The relative abundance of bacterial genera found to be significantly differentially abundant under metformin treatment, metformin dose, and/or hen age. (A) Relative abundance of the significant genera where “other” (in grey) shows the relative abundance of all other bacterial genera. (B) Inset showing the relative abundance of only the significant genera.**

**
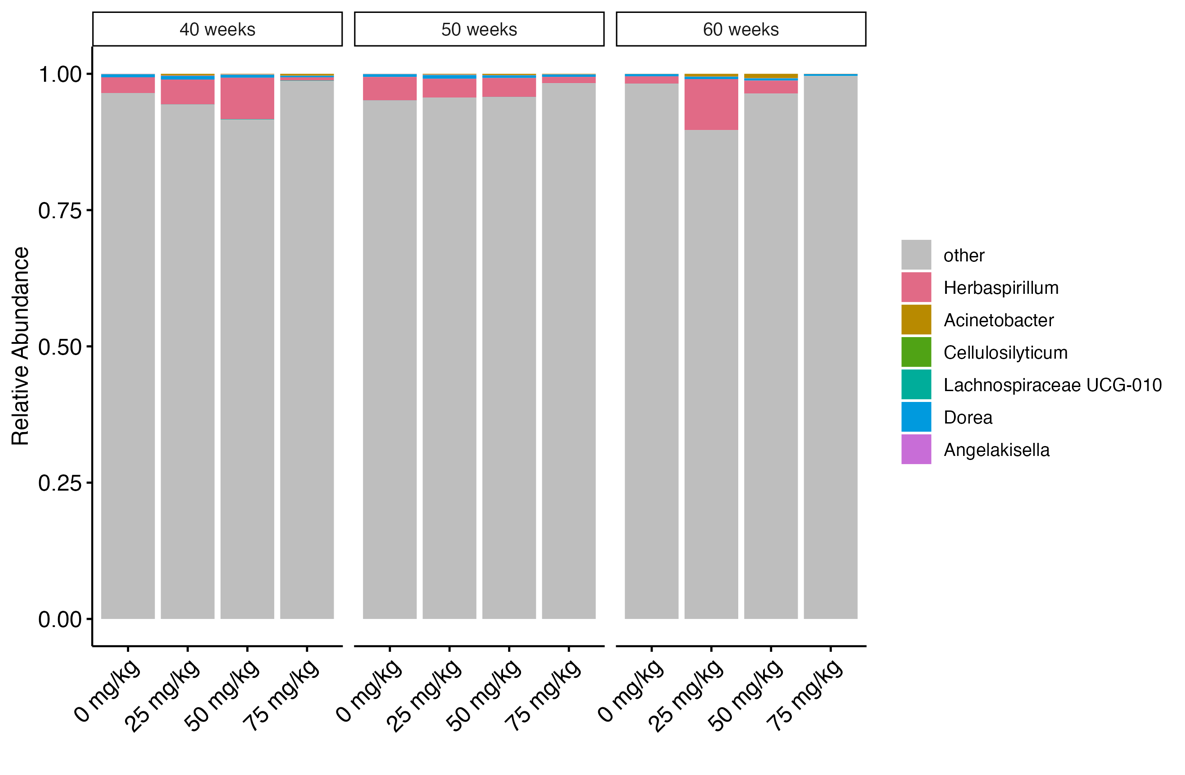
A.**

**
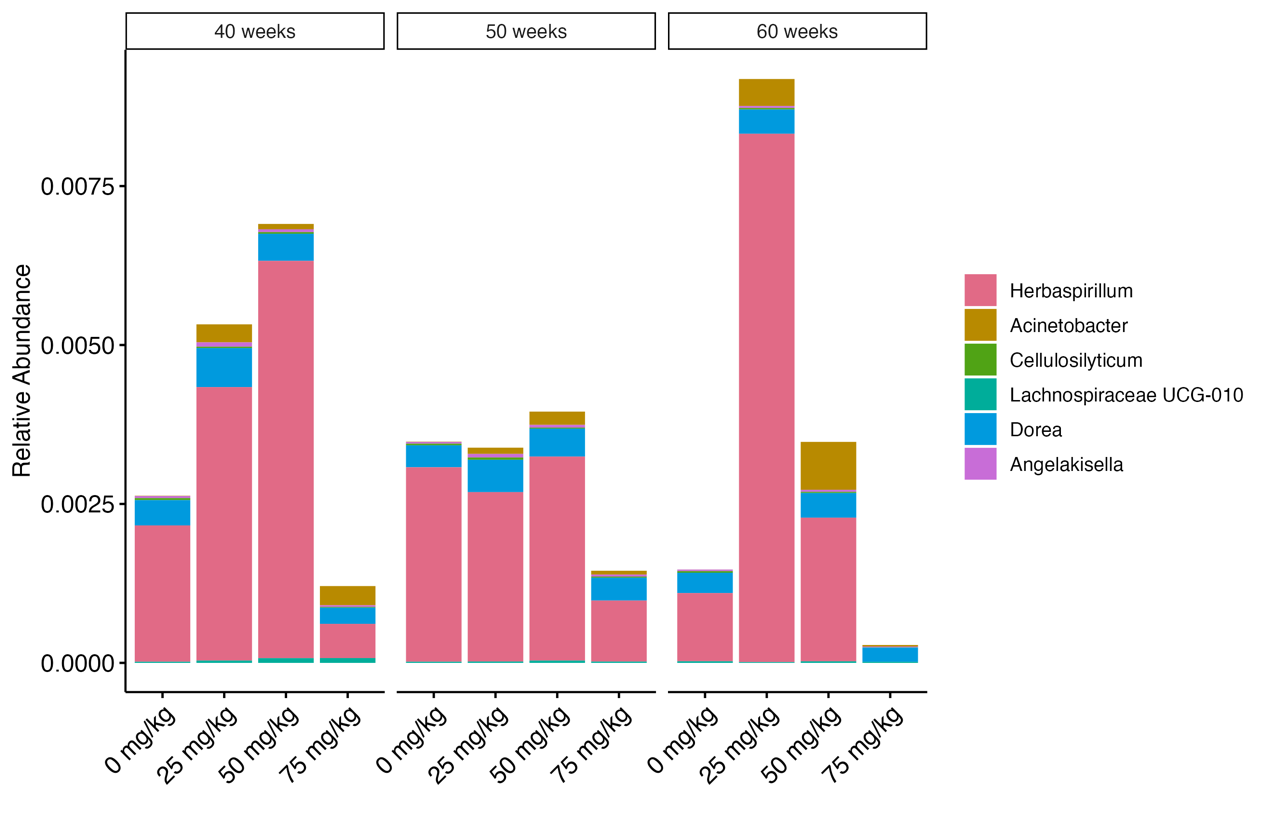
B.**
